# Obesity: Recent Advances and Future Perspectives

**DOI:** 10.3390/biomedicines13020368

**Published:** 2025-02-05

**Authors:** Miodrag Janić, Andrej Janež, Mohamed El-Tanani, Manfredi Rizzo

**Affiliations:** 1 Department of Endocrinology, Diabetes and Metabolic Diseases, University Medical Centre Ljubljana, 1000 Ljubljana, Slovenia; andrej.janez@kclj.si; 2 Faculty of Medicine, University of Ljubljana, 1000 Ljubljana, Slovenia; 3 School of Medicine, Promise Department of Health Promotion Sciences Maternal and Infantile Care, Internal Medicine and Medicinal Specialties, University of Palermo, 90133 Palermo, Italy; manfredi.rizzo@unipa.it; 4 College of Pharmacy, Ras Al Khaimah Medical and Health Sciences University, Ras Al Khaimah 11127, United Arab Emirates; eltanani@rakmhsu.ac.ae

As reported in the World Obesity Atlas 2024 by the World Obesity Federation, the projections for 2035 suggest that more than 1.77 billion people will be overweight, and 1.53 billion people will be affected by obesity. This will represent 54% of all adults worldwide. A significant proportion of these individuals will be in low- and middle-income countries. In 2024, more than 1 billion people live with obesity, and 1 of 8 deaths attributed to non-communicable diseases are driven by overweight or obesity, mostly due to diabetes, stroke, coronary heart disease, and cancer. In addition, the increasing incidence of this among young people is also alarming. It is pertinent to note that the global increase in incidence can be related to significant changes in diet patterns and a reduction in physical activity levels. The prevalence of obesity is not uniformly distributed, being more prevalent among individuals in socioeconomically deprived regions, older age groups, minorities, and people with disabilities. Furthermore, research has indicated that there is a direct association between obesity and socioeconomic status in low-to-middle-income countries; however, this relationship is inverse in high-income countries [1].

Obesity represents a chronic, complex, and multifactorial metabolic disease shaped by a convergence of genetic, biological, environmental, behavioral, sociocultural, and economic determinants. Obesity requires lifelong intervention. It poses numerous health-related risks [2]. Direct harm comes from excess adipose tissue and body weight itself, manifesting itself as osteoarthritis, back pain, obstructive sleep apnea, obesity hypoventilation syndrome, and asthma. Indirect harm is associated with metabolic dysfunctions related to obesity, which can present in various forms, including hypertension, dyslipidemia, cardiovascular diseases, type 2 diabetes, metabolic dysfunction-associated steatotic liver disease (MASLD), chronic kidney disease, gallbladder diseases, gout, as well as certain types of cancer and reproductive challenges. It is pertinent to note that people living with obesity are at higher risk for severe infections, as demonstrated during the COVID-19 pandemic, and have also been more exposed to post-COVID syndrome because of their heightened systemic inflammatory state and cardiometabolic derangements [3]. 

Indeed, people with obesity are more likely to require hospitalization and have a reduction in average life expectancy of 3 years, with severe obesity potentially reducing life expectancy by 8 to 10 years. Furthermore, people living with obesity experience higher incidences of depression and anxiety, have lower employment prospects and are subjected to increased discrimination and stigma [4]. 

In contrast, weight loss has been shown to confer various health benefits. Evidence suggests that a 5- to 15% reduction in body weight may significantly improve glycemic control and decrease the need for antihyperglycemic medications in individuals with type 2 diabetes. This weight reduction also contributes to improvements in blood pressure and a decrease in the need for antihypertensive treatment. Furthermore, it facilitates improvements in the lipid profile by reducing non-high-density lipoprotein (non-HDL) cholesterol and triglycerides while increasing HDL cholesterol levels. In addition, weight loss alleviates symptoms of obstructive sleep apnea and reduces the frequency of hypoxic episodes during sleep. A 10% weight reduction or more is correlated with the prevention of type 2 diabetes, the alleviation of osteoarthritis symptoms, and the improvement of physical condition, along with positive effects on MASLD [5]. It is notable that MASLD is an emerging problem in childhood, and the association between obesity, MASLD, and type 1 diabetes seems quite close [6].

Lifestyle interventions, consisting of reduced caloric intake and increased physical activity, constitute the cornerstone of obesity management. Nevertheless, lifestyle interventions are limited in their effectiveness concerning both the magnitude and durability of the weight loss achieved. Over the years, numerous pharmacotherapies have been developed to address obesity. However, it was not until the advent of glucagon-like peptide-1 (GLP-1) and glucose-dependent insulinotropic peptide/GLP-1 (GIP/GLP-1) receptor agonists that managing obesity became feasible and practical. These pharmacological agents have transformed obesity treatment, enabling the sustained achievement of favorable weight outcomes with an acceptable profile of side effects.

In December 2014, liraglutide at 3.0 mg daily became the first GLP-1 receptor agonist to receive approval from the Food and Drug Administration (FDA) in the United States for the treatment of obesity. This approval was based on the findings of the 56-week Satiety and Clinical Adiposity—Liraglutide Evidence in Nondiabetic and Diabetic Individuals (SCALE) study, which involved 3731 participants with a body mass index (BMI) exceeding 30 kg/m^2^ or at least 27 kg/m^2^ accompanied by dyslipidemia and/or arterial hypertension, but without diabetes. The study demonstrated that liraglutide treatment, when combined with lifestyle modification, resulted in an average reduction of 8.4 kg in body weight compared to a mean reduction of 2.8 kg observed in the placebo group. Furthermore, 63.2% of those treated with liraglutide achieved a minimum of a 5% reduction in body weight (compared to 27.1% in the placebo group), and 33.1% of those treated with liraglutide achieved at least a 10% reduction in body weight. However, this was accompanied by mild and moderate nausea and diarrhea [7]. This study facilitated subsequent research in the domain of GLP-1 receptor agonists for individuals without diabetes, with a particular emphasis on weight reduction. Subsequently, the Semaglutide Treatment Effect in People with Obesity (STEP) program started with the STEP-1 trial, which examined the effects of a weekly dose of 2.4 mg of semaglutide. In 2021, a 68-week course of semaglutide treatment in a study population analogous to that used for liraglutide produced a mean weight reduction of −14.9%, in contrast to a weight reduction of −2.4% observed in the placebo group. This finding translated into a mean reduction of −15.3 kg in the semaglutide group, compared to −2.6% in the placebo group. In total, 50.5% of the participants achieved at least a 15% reduction in their initial weight, compared to 4.9% in the placebo cohort. In addition, there was a notable improvement in cardiometabolic risk factors and reported physical functioning. Transient and mild-to-moderate nausea and diarrhea were the most frequently reported adverse effects within the semaglutide group, and 4.5% of people who received semaglutide discontinued treatment due to gastrointestinal side effects [8]. The STEP program includes a series of subsequent studies aimed at evaluating the effects and benefits of weight loss in diverse study populations. These studies report an average weight reduction ranging from 12% to 15% in people without type 2 diabetes. On the contrary, people with type 2 diabetes exhibit a lower degree of weight loss, as demonstrated in the STEP-2 trial [9]. Subsequently, comparable significant results were documented for the oral administration of semaglutide at a daily dose of 50 mg [10].

Tirzepatide, the first unimolecular GIP/GLP-1 receptor co-agonist for weight loss, was evaluated in the SURMOUNT program [11]. In the SURMOUNT-1 trial, 2539 individuals with overweight and obesity received tirzepatide at doses of 5, 10, or 15 mg, or a placebo, over a 72-week period. The mean percentage of weight reduction at the end of the trial was found to be −15.0%, −19.5%, and −20.9% for tirzepatide doses of 5, 10, and 15 mg, respectively, compared to −3.1% among the placebo group. In total, 85% of the participants experienced a weight reduction greater than 5% with a 5 mg dose, while 57% of the participants achieved a weight loss greater than 20% with a 15 mg dose. The incidence of adverse events that led to the discontinuation of treatment occurred in 4.3%, 7.1%, and 6.2% of participants who received 5, 10, and 15 mg of tirzepatide, respectively, compared to 2.6% in the placebo cohort [12]. The SURMOUNT program is expanding its evaluation to include diverse populations and assesses the implications of tirzepatide on weight-reduction outcomes. The effect of tirzepatide on weight loss in people with type 2 diabetes is like that observed in semaglutide treatment. However, it is significant to note that among people administered tirzepatide, the incidence of gastrointestinal adverse effects was comparatively lower than that observed with semaglutide despite a greater weight reduction. This is likely attributed to the actions of GIP in mitigating the centrally induced aversive response of GLP-1 [9].

The association between obesity and cardiovascular disease is unequivocal, as obesity represents a strong risk factor for cardiovascular disease. Furthermore, obesity is a propagator of several additional cardiovascular risk factors, namely arterial hypertension, dyslipidemia, type 2 diabetes, and sleep disorders. Evidence indicates that individuals with obesity, in the absence of other metabolic abnormalities, exhibit a 49% increased risk of coronary heart disease, a 7% increased risk of cerebrovascular disease, and a 96% increased risk of heart failure compared to people of normal weight without metabolic abnormalities over a median follow-up period of 5.4 years. This risk is further exacerbated by an increase in BMI and the presence of additional metabolic conditions, such as arterial hypertension, dyslipidemia, and type 2 diabetes, independent of sex [13]. The underlying mechanisms responsible for this increased risk include low-grade inflammation, demonstrated by the accumulation of circulatory inflammatory biomarkers, and insulin resistance. These factors contribute to the acceleration of endothelial dysfunction and atherosclerosis independently while also promoting the oxidation of low-density lipoproteins (LDLs) and modifying fibrinolysis and coagulation processes, thus creating a proatherogenic and prothrombotic environment [14]. 

Furthermore, excess adipose tissue directly impacts cardiac function through fat deposition in the myocardium and subsequent fibrosis, resulting in left ventricular diastolic dysfunction and heart failure with preserved ejection fraction (HFpEF). This association is noteworthy, as each increase in BMI by 1 kg/m^2^ corresponds to an increase in heart failure incidence by 5% in men and 7% in women, irrespective of other risk factors [15]. There is evidence that women have a better weight response to GLP-1 receptor agonists than men [16].

The definitive impact of semaglutide at a dose of 2.4 mg on the reduction in major adverse cardiovascular events (MACEs) has been supported by the Semaglutide Effects on Cardiovascular Outcomes in People with Overweight or Obesity (SELECT) trial. This trial enrolled 17,604 participants with a body mass index of 27 kg/m^2^ or greater and established atherosclerotic cardiovascular disease, excluding type 2 diabetes. The mean duration of follow-up in this trial was 39.8 months. A relative reduction in the risk of MACEs was observed by 20% in the group treated with semaglutide. These effects manifested early within the intervention cohort and were not solely attributable to weight loss. Individuals who received semaglutide demonstrated an average decrease of 37.8% in high-sensitivity C-reactive protein (hsCRP), along with significant reductions in systolic and diastolic blood pressure and a decrease of 15.6% in triglyceride levels. However, these results were accompanied by a discontinuation rate of 16.6% among semaglutide recipients, attributable to adverse effects [17]. Also, directly proportional to weight loss, there were improvements in symptoms and a reduction in systemic inflammation biomarkers in the STEP-HFpEF trial [18]. Furthermore, in a recent study, semaglutide administration at a dose of 2.4 mg was evaluated in a cohort of 101 overweight and obese individuals, characterized by a mean BMI of 36.2 kg/m^2^. These subjects were diagnosed with non-diabetic chronic kidney disease, chronic glomerulonephritis, and hypertensive chronic kidney disease, identified as the most prevalent etiologies. Participants exhibited an estimated average glomerular filtration rate (eGFR) of 65 mL/min/1.73 m^2^ and a median urinary albumin-to-creatinine ratio (UACR) of 251 mg/g. Within the cohort that received semaglutide, a notable reduction in UACR was observed by 52.1% after a treatment period of 24 weeks, independent of concurrent therapy with the SGLT-2 inhibitor, further supporting the efficacy of semaglutide in this clinical context [19]. Recently, the administration of semaglutide at a weekly dose of 2.4 mg was also associated with a significant reduction in body weight and alleviation of pain in individuals with obesity and radiologically confirmed moderate knee osteoarthritis [20]. Furthermore, semaglutide treatment contributed to improvements in liver fibrosis without exacerbating steatohepatitis, as well as resolution in steatohepatitis without the further deterioration of liver fibrosis compared to a placebo in individuals with metabolic dysfunction-associated steatohepatitis (MASH) and moderate-to-advanced liver fibrosis [21].

An investigation into tirzepatide’s effects on the reduction in morbidity and mortality in obese adults, titled SURMOUNT-MMO, is currently being conducted among individuals aged 40 years or older who have established cardiovascular disease or numerous cardiovascular risk factors. The main endpoint encompasses all-cause mortality, non-fatal myocardial infarction, non-fatal stroke, coronary revascularization, and heart failure events that require urgent medical consultations or hospital admissions (NCT05556512) [9]. 

A recent cross-sectional study conducted in the United States indicated that fewer than 6% of people with obesity but without diabetes who qualify for anti-obesity treatment received any form of treatment between 2022 and 2023. During the same period, there was a two-fold increase in the use of GLP-1 receptor agonists, accompanied by a 25.6% decrease in the frequency of metabolic bariatric surgeries. People who were still treated with bariatric surgery had more comorbidities, with 18.8% having more than four comorbid states compared to 8.2% of those treated with GLP-1 receptor agonists. These findings suggest a reduction in the prevalence of bariatric surgery at the same time as the advent of GLP-1 receptor agonists [22]. This study shows that due to their effectiveness, the GLP-1 and GIP/GLP-1 receptor agonists are overtaking the leading role in obesity management in the modern era while also reflecting the reluctance of prescribers and the logistical and financial inaccessibility of medications in eligible populations [23].

Therefore, based on all the benefits of GLP-1 receptor therapy, it is unsurprising that the recipients of this year’s Lasker-DeBakey Clinical Medical Research Award are Joel Habener, Svetlana Mojsov, and Lotte Bjerre Knudsen, in recognition of their pioneering work in the discovery and development of GLP-1-based therapy, which has significantly transformed obesity treatment [24].

“Overweight and obesity are defined as abnormal or excessive fat accumulation that presents a health risk”; this is a definition provided by the World Health Organization (WHO). The Obesity Medicine Association (OMA) characterizes obesity as a chronic, progressive, relapsing, and treatable multifactorial neurobehavioral disease in which an increase in body fat promotes the dysfunction of adipose tissue and abnormal physical forces of fat mass, culminating in adverse metabolic, biomechanical, and psychosocial health outcomes [25]. To effectively address obesity, it is imperative to adopt an approach aligned with both definitions, recognizing obesity as a complex multifactorial disease state that requires intervention. 

The prospects for managing obesity include the domain of personalized medicine, in which individualized treatment plans can be developed based on the genetic composition and clinical characteristics of individuals. However, lifestyle interventions will continue to be the cornerstone of obesity management alongside behavioral and cognitive behavioral therapy. Furthermore, it is imperative that individuals receiving anti-obesity pharmacotherapy participate in weight management programs to mitigate the potential loss of muscle mass associated with such interventions [26]. Furthermore, the development of biomarkers is underway to facilitate the early identification of obesity risk, alongside improvements in screening and prevention programs. The latter could result in long-term health benefits and improved quality of life, particularly during early childhood through school-based initiatives and public health campaigns that promote healthy eating and physical activity. The integration of artificial intelligence could enable the predictive modeling of obesity risk and disease progression, while current technologies, such as wearable devices to monitor physical activity and caloric intake and mobile phone applications for weight management and health coaching, could provide substantial benefits. Telemedicine could be used for the remote management of obesity care.

The development of new drugs is underway, aimed at targeting additional pathways involved in obesity. In the coming years, a significant number of effective pharmaceuticals are expected to be launched sequentially onto the market. These will consist of small molecule oral GLP-1 receptor agonists (danuglipron), unimolecular glucagon receptor (GCG)-GLP-1 receptor co-agonists (survodutide, mazdutide), as well as GCG-GLP-1-GIP receptor tri-agonists (retatrutide), distinct GLP-1/GIP receptor co-agonists, small molecule oral GLP-1/GIP receptor co-agonists, and combinations of long-acting amylin receptor (cagrilintide) with GLP-1 receptor agonists [9,27]. Available in both oral and injectable formulations, they will allow personalized choices from both a medical and logistic point of view. An expanded array of therapeutic options is expected to improve affordability with the objective of addressing the needs of a larger patient population. Bariatric surgery is likely to persist as a necessary intervention for people who need treatments beyond the capabilities of pharmacological approaches or for those who cannot tolerate medications. 

However, the issue of equitable distribution persists, particularly since, as initially noted, obesity represents a significant challenge in low-to-middle-income countries. Therefore, the availability of new and more affordable medications in these regions could promote more inclusive and equitable treatment opportunities on a global scale. Global health initiatives will be crucial to address disparities in obesity care and access to treatment while simultaneously promoting awareness and education on obesity.

## References

[1] (2024). World Obesity Atlas 2024.

[2] Janez A., Muzurovic E., Bogdanski P., Czupryniak L., Fabryova L., Fras Z., Guja C., Haluzik M., Kempler P., Lalic N. (2024). Modern Management of Cardiometabolic Continuum: From Overweight/Obesity to Prediabetes/Type 2 Diabetes Mellitus. Recommendations from the Eastern and Southern Europe Diabetes and Obesity Expert Group. Diabetes Ther..

[3] Rizvi A.A., Kathuria A., Al Mahmeed W., Al-Rasadi K., Al-Alawi K., Banach M., Banerjee Y., Ceriello A., Cesur M., Cosentino F. (2022). Post-COVID syndrome, inflammation, and diabetes. J. Diabetes Complicat..

[4] Patoulias D., Koufakis T., Ruza I., El-Tanani M., Rizzo M. (2024). Therapeutic Advances in Obesity: How Real-World Evidence Impacts Affordability Beyond Standard of Care. Pragmat. Obs. Res..

[5] Garvey W.T., Mechanick J.I., Brett E.M., Garber A.J., Hurley D.L., Jastreboff A.M., Nadolsky K., Pessah-Pollack R., Plodkowski R., Reviewers of the AACE/ACE Obesity Clinical Practice Guidelines (2016). American Association of Clinical Endocrinologists and American College of Endocrinology Comprehensive Clinical Practice Guidelines for Medical Care of Patients with Obesity. Endocr. Pract..

[6] Muzurovic E., Rizzo M., Mikhailidis D.P. (2022). Obesity and nonalcoholic fatty liver disease in type 1 diabetes mellitus patients. J. Diabetes Complicat..

[7] Pi-Sunyer X., Astrup A., Fujioka K., Greenway F., Halpern A., Krempf M., Lau D.C., le Roux C.W., Violante Ortiz R., Jensen C.B. (2015). A Randomized, Controlled Trial of 3.0 mg of Liraglutide in Weight Management. N. Engl. J. Med..

[8] Wilding J.P.H., Batterham R.L., Calanna S., Davies M., Van Gaal L.F., Lingvay I., McGowan B.M., Rosenstock J., Tran M.T.D., Wadden T.A. (2021). Once-Weekly Semaglutide in Adults with Overweight or Obesity. N. Engl. J. Med..

[9] Drucker D.J. (2024). Efficacy and Safety of GLP-1 Medicines for Type 2 Diabetes and Obesity. Diabetes Care.

[10] Knop F.K., Aroda V.R., do Vale R.D., Holst-Hansen T., Laursen P.N., Rosenstock J., Rubino D.M., Garvey W.T., Investigators O. (2023). Oral semaglutide 50 mg taken once per day in adults with overweight or obesity (OASIS 1): A randomised, double-blind, placebo-controlled, phase 3 trial. Lancet.

[11] Rangraze I., Patoulias D., Karakasis P., El-Tanani M., Rizzo M. (2024). Tirzepatide, a novel, dual glucose-dependent insulinotropic polypeptide/glucagon-like peptide-1 receptor agonist for the ongoing diabesity epidemic: The dawn of a new era?. Expert Rev. Clin. Pharmacol..

[12] Jastreboff A.M., Aronne L.J., Ahmad N.N., Wharton S., Connery L., Alves B., Kiyosue A., Zhang S., Liu B., Bunck M.C. (2022). Tirzepatide Once Weekly for the Treatment of Obesity. N. Engl. J. Med..

[13] Caleyachetty R., Thomas G.N., Toulis K.A., Mohammed N., Gokhale K.M., Balachandran K., Nirantharakumar K. (2017). Metabolically Healthy Obese and Incident Cardiovascular Disease Events Among 3.5 Million Men and Women. J. Am. Coll. Cardiol..

[14] Banerjee Y., Patti A.M., Giglio R.V., Ciaccio M., Vichithran S., Faisal S., Stoian A.P., Rizvi A.A., Rizzo M. (2023). The role of atherogenic lipoproteins in diabetes: Molecular aspects and clinical significance. J. Diabetes Complicat..

[15] Bozkurt B., Aguilar D., Deswal A., Dunbar S.B., Francis G.S., Horwich T., Jessup M., Kosiborod M., Pritchett A.M., Ramasubbu K. (2016). Contributory Risk and Management of Comorbidities of Hypertension, Obesity, Diabetes Mellitus, Hyperlipidemia, and Metabolic Syndrome in Chronic Heart Failure: A Scientific Statement From the American Heart Association. Circulation.

[16] Jensterle M., Rizzo M., Janez A. (2022). Weight response to GLP-1 receptor agonists: Why women do it better?. J. Diabetes Complicat..

[17] Lincoff A.M., Brown-Frandsen K., Colhoun H.M., Deanfield J., Emerson S.S., Esbjerg S., Hardt-Lindberg S., Hovingh G.K., Kahn S.E., Kushner R.F. (2023). Semaglutide and Cardiovascular Outcomes in Obesity without Diabetes. N. Engl. J. Med..

[18] Kosiborod M.N., Abildstrom S.Z., Borlaug B.A., Butler J., Rasmussen S., Davies M., Hovingh G.K., Kitzman D.W., Lindegaard M.L., Moller D.V. (2023). Semaglutide in Patients with Heart Failure with Preserved Ejection Fraction and Obesity. N. Engl. J. Med..

[19] Apperloo E.M., Gorriz J.L., Soler M.J., Cigarran Guldris S., Cruzado J.M., Puchades M.J., Lopez-Martinez M., Waanders F., Laverman G.D., van der Aart-van der Beek A. (2024). Semaglutide in patients with overweight or obesity and chronic kidney disease without diabetes: A randomized double-blind placebo-controlled clinical trial. Nat. Med..

[20] Bliddal H., Bays H., Czernichow S., Udden Hemmingsson J., Hjelmesaeth J., Hoffmann Morville T., Koroleva A., Skov Neergaard J., Velez Sanchez P., Wharton S. (2024). Once-Weekly Semaglutide in Persons with Obesity and Knee Osteoarthritis. N. Engl. J. Med..

[21] Novo Nordisk A/S: Semaglutide 2.4 mg Demonstrates Superior Improvement in Both Liver Fibrosis and MASH Resolution in the ESSENCE Trial. https://www.novonordisk.com/content/nncorp/global/en/news-and-media/news-and-ir-materials/news-details.html?id=171971.

[22] Lin K., Mehrotra A., Tsai T.C. (2024). Metabolic Bariatric Surgery in the Era of GLP-1 Receptor Agonists for Obesity Management. JAMA Netw. Open.

[23] Muzurovic E., Yumuk V.D., Rizzo M. (2023). GLP-1 and dual GIP/GLP-1 receptor agonists in overweight/obese patients for atherosclerotic cardiovascular disease prevention: Where are we now?. J. Diabetes Complicat..

[24] 2024 Lasker-DeBakey Clinical Medical Research Award: GLP-1-Based Therapy for Obesity. https://laskerfoundation.org/winners/glp-1-based-therapy-for-obesity/#achievement.

[25] Fitch A.K., Bays H.E. (2022). Obesity definition, diagnosis, bias, standard operating procedures (SOPs), and telehealth: An Obesity Medicine Association (OMA) Clinical Practice Statement (CPS) 2022. Obes. Pillars.

[26] Linge J., Birkenfeld A.L., Neeland I.J. (2024). Muscle Mass and Glucagon-Like Peptide-1 Receptor Agonists: Adaptive or Maladaptive Response to Weight Loss?. Circulation.

[27] Melson E., Ashraf U., Papamargaritis D., Davies M.J. (2024). What is the pipeline for future medications for obesity?. Int. J. Obes..

